# Sex-specificity in Surgical Stages of Lung Cancer in Young Adults

**DOI:** 10.2174/18743064-v17-230818-2022-20

**Published:** 2023-07-17

**Authors:** Mahdi Abdennadher, Mariem Hadj Dahmane, Sarra Zair, Hazem Zribi, Amina Abdelkbir, Imen Bouassida, Mouna Mlika, Imen Sahnoun, Amani Ben Mansour, Adel Marghli

**Affiliations:** 1 Department of Thoracic Surgery, Tunis El Manar University, Abderrahmen Mami Hospital Ariana, Tunisia; 2 Department of Pathology, Tunis El Manar University, Abderrahmen Mami Hospital, Tunisia; 3 Department of Pneumology Pavillon D, Tunis El Manar University, Abderrahmen Mami Hospital Ariana, Tunisia; 4 Department of Pneumology Pavillon C, Tunis El Manar University, Abderrahmen Mami Hospital Ariana, Tunisia

**Keywords:** Lung cancer, Young male, Young female, Incidence, Histologic type, Lung surgery, Prognosis

## Abstract

**Background:**

Young Patients with lung cancer represent a distinct subset of patients with this neoplasm. Young International studies show increased lung cancer rates in females, while the incidence in males continues to decline. There is evidence to suggest that this trend recurs in younger patients. We studied the effects of gender differences on the incidence of surgical stages of lung cancer in young adults and its mortality rate.

**Methods:**

This study is a retrospective review (2010-2020) of young adults (aged under 45 years) with surgical-stage of lung cancer. We calculated female-to-male differences in incidence rate ratios, tumor characteristics, surgical management, and survival. Cumulative survival curves were generated by the Kaplan-Meier method.

**Results:**

We examined 46 men and 24 women, under 45 years. Female patients were diagnosed at earlier stages. The proportion of stage IA disease was significantly higher in women than in men (46% *versus* 13%, respectively) (p=0.03). Women were more likely never smokers (42% *versus* 83%, p=0.02). A histologic subtype, females were more likely to have typical carcinoid tumors (13.54% *versus* 10.21% for males) (p>0.05). The largest histological type in men was adenocarcinoma (25.53% *versus* 4.16%, p>0.05). All the patients were operated. Three men had neoadjuvant chemotherapy and one was operated on for cerebral oligometastatic before his chest surgery. Adjuvant chemotherapy was given to 7 women and 21 men. Despite the small number of postoperative complications in our study (n= 8, 11.2%), the male sex was significant in predicting this complication (p<0.05). The mortality rate was 1.4%. The 5-year overall survival rates were 84% in men and 87% in women.

**Conclusion:**

Our study identified sex differences in the incidence and mortality rates for surgical lung cancers in young adults, but the biological and endocrine mechanisms implicated in these disparities have not yet been determined.

## 1 INTRODUCTION

Lung cancer is mainly a neoplasm of the elderly, with a peak of incidence between the sixth and seventh decades of life, only 5 to 10% of non-small cell lung cancer (NSCLC) are diagnosed in individuals younger than 50 years of age [1]. Lung cancer is the leading cause of mortality worldwide among men and the second most common cancer among women [2, 3]. In Tunisia, both incidence and mortality from lung cancer continue to increase sharply and constitute a serious threat to public health. International data demonstrate increased rates of lung cancer in women, while the incidence in men continues to fall [3]. However, previous studies have reported converging lung cancer rates between sexes [4].

This single-center study was designed to define the characteristics of lung cancer in surgical stages and its incidence rates in young women *versus* young men in 45-year-old or younger in our population. Our overview will help to expand our understanding of this subgroup of lung cancer and provide good prevention and improved prognosis.

## 2 MATERIALS AND METHODS

### 2.1 Study Population

This study is a retrospective review. From January 2010 to December 2020, 71 adults aged between 6-45 years operated for lung cancer confirmed by pathological examination in the thoracic surgery department of Abderrahmen Mami University Hospital were reviewed. The following data were retrieved: age; sex; family history; medical history; personal history (including smoking); The international TNM (tumor-node-metastasis) system, 8th edition, was used to define tumor stage; histological type; surgical approach; interventions; post-operative complication; neoadjuvant and adjuvant treatment; and overall survival (OS).

Overall survival was defined as the interval from diagnosis to death or the latest follow-up. The end date for the last follow-up was December 2020.

### 2.2 Statistical Analysis

A comparison between the characteristics of lung cancer and its incidence rates in young women *versus* young men was carried out using SPSS software. Cumulative survival curves were generated by the Kaplan-Meier method. *P*-values < 0.05 were considered statistically significant.

## 3 RESULTS

### 3.1 Patients Characteristics

#### 3.1.1 Age and Sex

A total of 71 young patients (46 males (66%) and 24 females (34%)) were operated for lung cancer from January 2010 to December 2020 in Abderrahmen Mami University Hospital from a total of 2243 (3.16%) patients operated for lung cancer of all ages in this period. The sex ratio was 2. The median age was 37 years (range 6-45), 73% of patients were 35 to 45-year-old and 23% were younger than 35 years old. There was no significant difference between age group-related gender (*P*=0.9) (Table **1**).

#### 3.1.2 Smoking

Forty nine patients were smokers, while 22 had no history of smoking. Among the smokers, 40 patients were with ≥ 15 years of smoking history.

We also calculated the female-to-male ratios of the prevalence of smoking to assess whether the smoking prevalence differed significantly between men and women. In our study, smoking prevalence was considerably higher among men (83%) than among women (42%) (p=0,02).

#### 3.1.3 Comorbidity

Family history was unremarkable in 80% of patients. Three patients had a familial history of cancer (n=2: lung cancer, n=1: thyroid cancer).

Two women had a medical history (8.3%) *versus* three men (6.4%). Two patients had a history of hypertension, 2 had a history of diabetes, and 1 had a history of dyslipidemia. Two patients were treated for chronic obstructive pulmonary disease.

#### 3.1.4 Histologic Types and Staging

Adenocarcinoma was the predominant histologic type in our study (39%, n = 28), followed by typical carcinoid (32%, n = 23), squamous carcinoma (8,4%, n = 6), and atypical carcinoid (2,8%, n = 2). Infrequent pathologic subtypes including mucoepidermoid carcinoma, adenoid cystic carcinoma, large cell carcinoma, Blastoma, and sarcomatoid carcinoma were presented in Table **2**. Analysis of the histologic data indicated that there were no significant differences in the distribution of type between the sexes. The largest histological type in females were typical carcinoid tumors and adenocarcinoma. The male group had more adenocarcinoma than the typical carcinoid. p TNM staging is presented in Table **3**.

### 3.2 Treatment and Outcome

Three patients had neoadjuvant chemotherapy (3 men) and one male patient was operated on for cerebral oligometastatic before his chest surgery. Adjuvant chemotherapy was given to 28 patients; 7 women and 21 men.

The operative approaches were posterolateral thoracotomy (n= 60, 84.5%), lateral thoracotomy (n= 5, 7%), and Video-assisted thoracotomy (VATS) (n=6, 8.5%).

The operative procedures were extended lobectomy (lobectomy + chest wall) (n=4, 5.6%), standard lobectomy (n= 43, 60.5%), sleeve lobectomy (n=2, 2.8%), bilobectomy (n=10, 14%), pneumonectomy (n=9, 12.6%), and segmentectomy (n=3, 4.22%). Systemic lymph node dissection was performed in all the patients.

The relationship between gender and surgical procedures is presented in Table **4**.

The mean postoperative hospital stay was 6 days, with a range of 2 days.

Despite the small number of postoperative complications in our study (n= 8, 11.2%), the male sex was significant in predicting complications (*p* <0.05). The frequency of postoperative complications was higher after lobectomy (n=7, 10%) than after pneumonectomy (n=0) and bilobectomy (n=1, 1.4%). Postoperative bleeding and empyema leading to re-exploration were seen in 2 patients after lobectomy.

The mortality rate was 1.4% (a man) showed after lobectomy. The number of deaths was too low to identify any significant risk factors. The relationship between patient characteristics or operative factors and postoperative complication are presented in Table **5**.

### 3.3 Survival Analysis

**Fig. (1):**
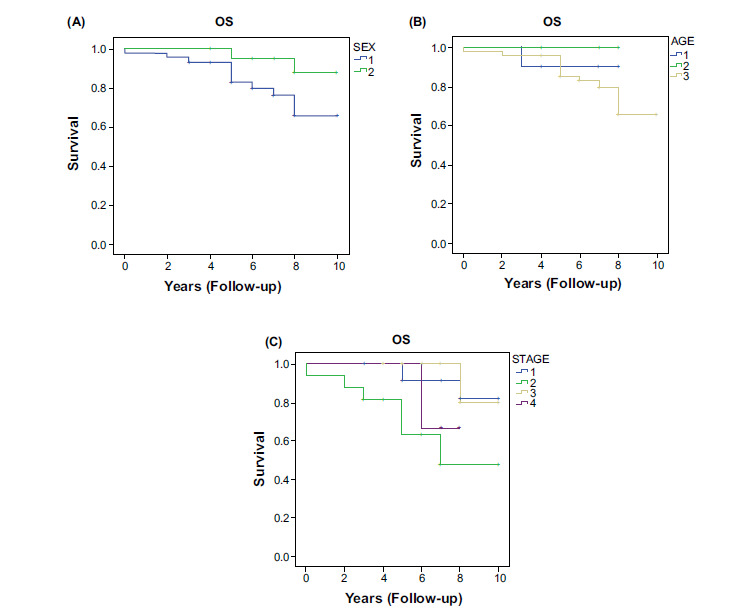
(**A**). influence of sex on overall survival (OS) in young adults with lung cancer in operated stage (1: Male, 2: female). (**B**). influence of age on overall survival (1: < 25 YEARS, 2: 25-35 YEARS, 3: >35 YEARS). (**C**). influence of stage on overall survival (1: Stage I, 2: Stage II, 3: Stage III, 4: Stage IV).

The actuarial survival rate of all patients at 3 and 5 years was 95% and 89%, respectively; 12 patients had died at the time of this study. Sex was associated with overall survival. The overall survival rate for females (median= 7 years) was superior to that of males (median = 5 years) (Fig. **1A**). These results can be explained by the different types of cancer between the sex and their evolution, for example, Females have a high number of carcinoids whereas males had a higher number of adenocarcinoma, both of which have different prognoses. Also, females have fewer postoperative complications than males. All these prognostic factors influenced the overall survival.

The median survival rates for patients aged between 35 and 45 years and patients under 25 years and between 25 and 35 years, were undefined, 6 years, 7 years, and 5 years respectively. The stage was another important prognostic factor for overall survival. Surprisingly, patients with stage II disease had much shorter overall survival than those with stage I-III (p > 0.05) (Fig. **1C**).

To conclude, our study showed that the female gender was associated with more favorable short- and long-term outcomes after surgery for lung cancer.

Kaplan-Meier survival curves according to sex, age, and tumor stage are presented in Fig. (**1**).

## 4 DISCUSSION

Research on lung cancer in the young population is attracting considerable attention with rapid increases in the morbidity and mortality attributable to lung cancer [2]. Studies suggest that lung cancer in younger patients has distinct characteristics, particularly with respect to patient gender, smoking status, and tumor morphology [4]. Not only are the types of lung cancer different between men and women, the general risk of developing this disease appears to vary between the sexes. Aside from sex, most cases of lung cancer occur in patients in the sixth and seventh decades of life, but research from Europe and Asia (Japan and China) have demonstrated a trend of increasing incidence of lung cancer in young adults [2].

The aim of the study was to evaluate sex differences among young patients under the age of 45 years operated for lung cancer regarding risk factors, histological type, treatment modalities, adverse events, and survival.

Among all kinds of cancers, lung cancer remains the leading type in both incidence and mortality, with 2.1 million new cases and 1.8 million deaths in 2018 [5-7]. According to a study conducted in China, 2019,around 1.4% of lung cancer occurs in people under 35 years [2]. The definition of young adults with regard to lung cancer is uncertain and generally ranges from 40-50 years [2]. One of the studies in the UK reported that young adults (18–39 years) accounted for 0.5% of non-small cell lung cancer from 2004–2011 [8, 9], and another in the USA detected an incidence rate of 1.16% in a young population (18–40 years) [10]. One of the ways in which lung cancer differs in young people related to the gender is that young women are affected more by the disease than young men [11]. In contrast, the ratio seen in the larger population of people with cancer is 57.8% male to 45.9% female [12]. This finding is consistent with our study showing that younger patients with NSCLC are more likely to be male (66% males and 34% females).

The predominant type is the NSCLC which accounts for 85% of all cases [13]. Adenocarcinoma is the most common subtype that we find in young and even older patients. The distinction of cancer type is important in the clinic, not only for staging but also for treatment and prognosis. Initially, a difference between sexes in the incidence of lung cancer histological types was reported. Females are more likely to develop adenocarcinoma rather than squamous cell carcinoma or large cell lung cancer [6, 14]. Adenocarcinoma accounted for more than 50% of female cases in 2010 [15]. In contrast, in males, squamous cell carcinoma is the most common histologic subtype [6]. Analysis of the histologic data in our study indicated that the largest histological type in females was typical carcinoid tumor (n=13, 54%), and in males was adenocarcinoma (n= 25, 53%).

The case-control study by Brownson *et al*. examined the relationship between smoking and lung cancer by gender and histologic type. The result showed that the relative risk associated with ever-smoking, and the level of smoking was consistently higher in women than men for all lung cancer combined (ever-smoking odds ratios: 12.7 for females and 9.1 for males) [6]. Harris *et al*. concluded through a case-control study that women are more at risk than men for a given level of smoking (relative risk = 1.7) [6, 16]. Another cohort study in Iceland showed that the risk of lung cancer was more higher among female smokers than male smokers [6, 17].

While roles of sex hormones in other types of cancers affecting females or males have been identified and described, little is known about the influence of sex hormones in lung cancer. One potential mechanism identified to date is the synergism between estrogen hormone and some tobacco compounds, and oncogene mutations, in inducing the expression of metabolic enzymes, leading to enhanced formation of reactive oxygen species and DNA adducts, and subsequent pulmonary carcinogenesis [15]. The elevation of estrogen circulating levels in women together with the reduction of DNA repair capabilities, cause women more vulnerable to the cancer-causing effect of tobacco [18]. Researchers have shown that Anti-estrogen treatment strategies can decrease tumor size, growth, and cell proliferation which may contribute to improved patient outcomes [19]. Regarding progesterone, this hormone is involved in cell proliferation and lung disease emerging evidence has revealed an active role in lung carcinogenesis. Some studies have revealed that progesterone receptors are present commonly in non-tumor lung tissues compared with cancer tissue [20]. The role of male sex hormones in lung cancer has also been reported in the literature. The androgen receptor, which is expressed mostly in pneumocytes and lung epithelium of male patients, is known to be an active player in lung cancer pathogenesis [21]. The role of hormone replacement therapy used in postmenopausal women was presented in Table **6**.

Studies have shown that increasing the resection rates for lung cancer improves cancer survival. The literature contains conflicting data about the prognosis of young patients with lung cancer. In 2010, on analysing the SEER database (Surveillance, Epidemiology, and End Results), Subramamian *et al*. [10] found that younger age was clearly associated with reduced mortality rate due to NSCLC. On the contrary, in a case-control study by Bryant *et al* [26], patients younger than 45 years showed a 5-year overall survival of 51%, statistically worse than the 5-year overall survival of 62% in patients older than 45 years (p = 0.04). This is true, that younger adults are typically healthier with few morbidities and receive more complete and aggressive treatment that could explain better survival [1, 2]. However, at the same time, they underestimate symptoms and thus delay medical visits. To sum up, this difference could be related to the delay in diagnosis and treatment, but also to the different genetic and molecularcharacteristics of lung cancer in young patients, especially those who were never smokers.

The second aspect we investigated is thedifference between female and male patients with lung cancer is the prognosis which may differ. A recent Spanish nationwide prospective cohort study showed that the perioperative profile is better in women than in men (less often current or ex-smokers, had better lung functions and fewer comorbidities) [27, 28]. This study also showed that lower FEV1 is associated with increased cardiovascular and all-cause mortality [27, 28]. These findings imply that women are overall better candidates for surgical treatments [28] which could be related to a long-term survival as well. Women, in a Norwegian population-based study, were an independent favorable prognostic factor regardless of stage, age, type of operation, or histology [29]. In another study, in surgically treated early-stage (I and II) lung cancer, patients, age, gender, tumor size, and type of surgery are the important predictors of survival [30].

Our results showed that the female gender was associated with more favorable short- and long-term outcomes after surgery for lung cancer. Male sex was significant in predicting postoperative complications (*p* <0.05). In Kaplan-Meier analyses, the median survival rate was about 7 years in the women group and 5 years in the men group.

Our study has several limitations. First, it was a single-center retrospective investigation. Further, a relatively small proportion of our patients underwent genetic assessment. In the future, a large-scale and deeper investigation of this topic will be initiated and prospective studies will be required, to clarify the biological and genetic variance of lung cancer in younger males and females.

## CONCLUSION

Lung cancer in younger patients is a distinct entity that predominantly presents as adenocarcinoma, with late-stage at onset, and a higher frequency of gene alteration, relative to older patients. Much recent research has shown a trend of increasing the incidence of lung cancer in young adults. It remains unknown, how sex influences this cancer risk, treatment decisions, and outcomes. However,evidence of specific differences in presentation, smoking risk, and mutational burden supports the need to better leveragethese gender-associated differences to improve further detection, diagnosis, and systemic regimens to advance theoverall care strategy for young people with lung cancer.

## Figures and Tables

**Table 1:** The incidence of operated lung cancer grouped by gender and age group.

**Age Gender**	**Male (N,%)**	**Female (N,%)**
**< 25 Years (group 1)**	7 (15%)	4 (16%)
**25-35 Years (group 2)**	3 (6%)	5 (21%)
**>35 Years (group 3)**	37 (79%)	15 (63%)
**Total**	47 (100%)	24 (100%)

**Table 2:** Histological type of lung cancer grouped by gender and age.

**Gender Histologic Type / Age**	**Female**	**Male**
**Adenocarcinoma**	**4**	**24**
**< 25 years**	-	1
**25-35 years**	-	2
**35-40 years**	-	3
**>40 years**	4	19
**Blastome**	**-**	**1**
**6 years**	-	1
**Atypical Carcinoid**	**2**	**-**
**23 years**	1	-
**30 years**	1	-
**Typical Carcinoid**	13	10
**< 25 years**	2	3
**25-35 years**	4	1
**35-40 years 36**	2	5
**>40 years**	5	1
**Large Cell Carcinoma**	-	1
**36 years**	-	1
**Adenoid Cystic Carcinoma**	2	-
**37 years**	2	-
**Sarcomatoid Carcinoma**	-	4
**40 years**	-	1
**45 years**	-	3
**Choriocarcinome**	1	-
**38 years**	1	-
**Squamous Carcinoma**	1	5
**< 25 years**	-	1
**25-35 years**	-	-
**35-40 years**	1	2
**>40 years**	-	2
**Mucoepidermoid Carcinoma**	1	1
**13 years**	-	1
**24 years**	1	-

**Table 3:** Tumor clinical staging grouped by gender.

**Gender** **Stage**	**Female**	**Male**	***P*-value**
IA		
	11	6	P=0,03
IB	5	12	
IIA	1	8	P>0,05
IIB	4	9	
IIIA	2	9	P>0,05
IIIB	-	1	
IV	1	2	P>0,05
Overall total	24	47	-

**Table 4:** Operative approach and procedures presented by gender.

-	**Female**	**Male**	***p* value**
**Operative Approach**			-
Posterolateral thoracotomy	19	41	p > 0.05
Lateral thoracotomy	2	3	
VATS	3	3	
**Operative Procedures**			p > 0.05
Lobectomy	14	29	
Extended lobectomy	0	4	
Sleeve lobectomy	1	1	
Pneumonectomy	3	6	
Bilobectomy	5	5	
Segmentectomy	1	2	

**Table 5:** Relationship between patient characteristics or operative factors and postoperative complication.

-	**Postoperative Complications**		***p*-value**
	**Present**	**Absent**	
**Gender**			**< 0.05**
**Male**	8	39	
**Female**	0	24	
**Smoking History**			**< 0.05**
**Present**	7	42	
**Absent**	1	21	
**Staging**			> 0.05
**Stage I**	3	31	
**Stage II**	5	17	
**Stage III**	0	12	
**Stage IV**	0	3	
**Operative Procedure**			**< 0.05**
**Lobectomy or more**	8	60	
**segmentectomy**	0	3	

**Table 6:** Role of hormone replacement therapy in female lung cancer.

**Study/Refs**	**Study Type**	**Sample Size**	**Conclusion**
Chao *et al*., 2019 [22]	Retrospective cohort study	968,440	The use of hormone replacement therapy is associated with a decreased risk of lung cancer in women.
Titan *et al*., 2019 [23]	Randomized control trial	75,587	Hormone replacement therapy has a protective effect on lung cancer development among women
Jeon *et al*., 2020 [24]	Cross-sectional study	4,775,398	No statistically significant association was found between reproductive factors and the risk of lung cancer in postmenopausal women
Abdel-Rahman, 2020 [25]	Randomized control trial	77,911	Prior exposure to hormone replacement therapy is protective against lung cancer development and seems to be protective against death from lung cancer.

## Data Availability

Not applicable

## LIST OF ABBREVIATIONS

NSCLC: Non-small Cell Lung Cancer
OS: Overall Survival
TNM: Tumor, Node, and Metastasis
VATS: Video Assisted Thoracic Surgery
